# Focusing on Quality Patient Care in the New Global Subsidy for Malaria Medicines

**DOI:** 10.1371/journal.pmed.1000106

**Published:** 2009-07-21

**Authors:** Suerie Moon, Carmen Pérez Casas, Jean-Marie Kindermans, Martin de Smet, Tido von Schoen-Angerer

**Affiliations:** 1 Giorgio Ruffolo Doctoral Research Fellow, Sustainability Science Program, Center for International Development, Kennedy School of Government, Harvard University, Cambridge, Massachusetts, United States of America; 2 Campaign for Access to Essential Medicines, Médecins Sans Frontières, Geneva, Switzerland; 3 Médecins Sans Frontières, Brussels, Belgium

## Abstract

Tido von Schoen-Angerer and colleagues discuss the new Affordable Medicines Facility for malaria (AMFm), which subsidizes and facilitates access to artemisinin-based combination therapy, and what mechanisms are needed to ensure it stays focused on quality patient care.

## Introduction

Almost 40 years ago, Chinese scientists rediscovered the near-miraculous potency of artemisinin derivatives against malaria. Today, we are approaching a decade since the World Health Organization (WHO) recommended that artemisinin-based combination therapies (ACTs) replace older antimalarials rendered ineffective by resistance [1]. Yet the global malaria community—researchers, governments, international organizations, funding agencies, nongovernmental organizations, and activists—has collectively failed to provide widespread access to this treatment and to minimize the threat of resistance. The evidence is sobering:

- Although nearly all endemic countries have adopted ACTs as first-line therapy for *Plasmodium falciparum* malaria, access on the ground remains dangerously low. In recent household surveys from 18 African countries, on average only 3% of febrile children under five years received an ACT, while only 38% had access to other antimalarials. African children comprise 85% of global malaria deaths [2].
- The parasite is demonstrating decreased sensitivity to artemisinin in Cambodia, where use of artesunate monotherapy and substandard artemisinin-based drugs remains common [3]–[5]. If artemisinin-resistant strains of *P. falciparum* emerge and spread, they would weaken the last effective antimalarial we have.

Therefore, the Affordable Medicines Facility–malaria or AMFm (see Box 1) is a welcome step toward improving access to this lifesaving treatment. Funding commitments from UNITAID and the UK Department for International Development, along with the Global Fund to Fight AIDS, Tuberculosis and Malaria (GFATM) decision to host the facility, have launched an ambitious global subsidy on ACTs into operation. By lowering the price of ACTs, the AMFm may broaden access in the public sector. However, since many governments already receive GFATM support to purchase ACTs for public use, the AMFm's most dramatic impact is likely to be on prices in the private sector. (The term “private sector” here refers to for-profit entities and can denote a wide range of drug outlets, from small rural kiosks to regulated urban pharmacies and private clinics.) Approximately half of suspected malaria patients seek care outside the public sector in the WHO African and Western Pacific regions, and up to 78% in the Southeast Asian region [2].

**Box 1. The Affordable Medicines Facility–malaria (AMFm)**

The Affordable Medicines Facility–malaria (AMFm), a new global health initiative, aims to address inadequate access to ACT for treating *P. falciparum* malaria by subsidizing producer prices. First proposed in 2004 [33], the facility is expected to begin operating in late 2009. Resistance to older antimalarial drugs such as CQ or SP is now widespread, making ACTs the most effective treatment for *P. falciparum*, the deadliest variant of malaria; however, ACTs cost 10 to 20 times as much as CQ or SP. The AMFm aims to lower end-user prices to the level of older antimalarials in order to save lives by making ACTs more affordable and to delay resistance to artemisinin derivatives by driving artemisinin monotherapy and substandard antimalarials out of the market. The AMFm is hosted by the GFATM, and 11 countries have been invited to participate in the initial phase: Benin, Cambodia, Ghana, Kenya, Madagascar, Niger, Nigeria, Rwanda, Senegal, Tanzania, and Uganda.

**Figure 1:**
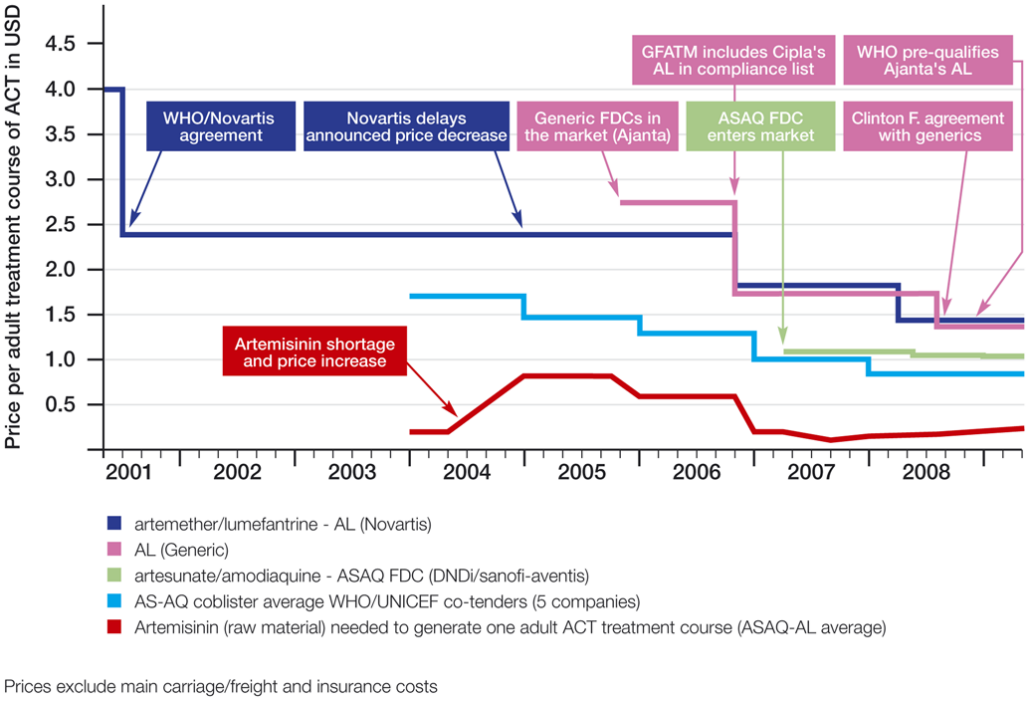
Evolution of prices for ACT adult treatment. Sources of prices are as follows: Artemether/lumefantrine (Novartis): Publicly announced prices by Novartis September 29, 2006 [34]; Novartis April 23, 2008 [35], and WHO May 16, 2007 [36]. Artemether/lumefantrine (generic): Prices quoted to MSF (manufacturer: Ajanta) followed by prices announced by Clinton HIV/AIDS Initiative (manufacturers: Cipla, IPCA) July17, 2008 [37]. Artesunate/amodiaquine FDC (DNDi/sanofi-aventis): Prices paid by MSF Logistique to manufacturer (MSF internal data). See also sanofi-aventis/DNDi announcement [38]. Artesunate/amodiaquine co-blister average WHO/UNICEF co-tenders: average price from five tenders [39]. Artemisinin (raw material): [40]. The artemisinin raw material considered here as the quantity needed for production of one adult treatment course with artesunate/amodiaquine is 613 mg and 839 mg with artemether/lumefantrine (in both cases with 5% lost during manufacturing).

ACT prices have significantly decreased in recent years, mainly due to competition (see Figure 1); however, they are still costlier than most antimalarials. ACT production incorporates a relatively expensive extraction, purification, and derivatization process, the cost of the companion drug (e.g., amodiaquine), and the cost of co-formulation for fixed-dose combinations (FDCs). Thus, a subsidy is warranted to bring ACT prices down to the level of other antimalarials, at least until semi-synthetic artemisinin production is available at adequate volume and low cost, which is not expected before 2012 [6].

The AMFm is both promising and ambitious. However, as the first major global initiative of its kind, its precise impact and consequences are still unknown. As the AMFm prepares for its first phase of implementation, it is critical to recognize areas that require further attention, where additional research is urgently needed, and how countries can best take advantage of the opportunities it may offer.

In the first section we propose policies to improve patient care. We then briefly suggest measures that could improve AMFm implementation. Finally, we discuss the implications of our analysis for calibrating support for the public and private sectors.

## I. Focusing on Quality Patient Care

The AMFm should adopt policies that will enhance quality patient care, including exclusively funding FDCs, withholding support for ineffective combinations, and supporting wider adoption of rapid diagnostic tests (RDTs).

By reducing pill count and making it impossible for the patient to take artemisinin monotherapy, FDCs can facilitate patient adherence and reduce the risk of resistance. The advantages of FDCs have been demonstrated in several disease areas, including tuberculosis and HIV/AIDS [7]–[9]. Furthermore, problems with inappropriate use of ACT co-blisters have been documented, such as removal of artesunate from co-blisters for sale as monotherapy [3]. In the near future, there are likely to be two or more WHO-prequalified (WHO-PQ) FDCs for two key drug combinations (artemether/lumefantrine and artesunate/amodiaquine); in addition, a pediatric FDC of artesunate/mefloquine is in the WHO-PQ assessment process [10]. (The new GFATM quality assurance policy, which will apply to the AMFm, allows the purchase of products still undergoing WHO-PQ assessment under certain conditions [11].) The growing number of manufacturers provides a viable way for the AMFm to subsidize exclusively FDCs from the outset, while encouraging generic competition through a transparent process. If there are insufficient FDCs available, temporary use of co-blisters could be acceptable. By endorsing the exclusive use of FDCs, the AMFm would send a clear signal to manufacturers to invest in the rapid development of such formulations, including all necessary quality, safety, and efficacy considerations. Unfortunately, while the AMFm Guidelines for Applications allow FDC purchase, they do not limit or phase out co-blisters [11].

Furthermore, the AMFm should not support the purchase of a particular combination when there is already significant resistance to the partner drug. Specifically, the combination of artesunate/sulfadoxine-pyrimethamine (SP) may not be suitable for use in countries where SP resistance or the risk of its emergence is relatively high. Current AMFm guidelines are silent on this issue [11].

In addition, the AMFm should support wider use of RDTs to improve the quality of patient care and minimize unnecessary ACT use [12]. Médecins Sans Frontières (MSF) has used RDTs in its field projects since 2002, and has found that a surprisingly high proportion of suspected malaria cases in sub-Saharan Africa were, in fact, not malaria. For example, negative outcomes comprised 35% of tested patients in Bo District, Sierra Leone, where transmission is high year-round, and 40% in Bongor District, Chad during the malaria high season [13]. Accurate diagnosis offers several benefits conducive to AMFm's broader objectives: if RDTs rule out malaria, providers can seek and treat the true cause of fever (or other symptoms), ultimately preventing deaths from other life-threatening illnesses [14],[15]; patients' perceptions of the efficacy of ACTs are likely to improve if they only take ACTs when they will be effective; and finally, the risk of resistance to partner drugs (particularly those with a long half-life) is likely to decrease with less “drug pressure” in the population—that is, if only patients that actually have malaria are taking ACTs. The mere availability of RDTs is not sufficient to guarantee improved outcomes; for example, it is not unusual for health care providers to prescribe an antimalarial after a negative test result [16]–[18], and quality may be compromised due to poor manufacturing or prolonged heat exposure [19],[20]. Thus, careful provider training and independent product testing, as recently conducted by WHO and its partners [21], are also important to maximize the potential benefits of RDTs.

While there is some experience using RDTs in the public sector, including through malaria village workers (MVWs) [3],[22],[23], the feasibility and/or best strategies to promote RDTs in the private sector remain unclear. Challenges to implementing RDT use in the private sector are many, including incentives for private vendors to overprescribe drugs or RDTs to maximize sales, the necessity of setting prices correctly to motivate their use, the lack of trained staff to administer the RDT, and concerns about the safety of taking RDT blood samples outside a clinical setting. The AMFm allows countries to use expected savings from lower ACT costs in current GFATM grants to expand RDT use as a supporting intervention; countries should take advantage of this opportunity. Governments should also request funds for operational research to improve RDT use in the public sector and explore possibilities for RDT provision in the private sector in order to strengthen the evidence base for the second phase of AMFm.

While decreasing ACT prices is a critical step, achieving widespread access to effective malaria treatment will require an approach to care that goes well beyond an affordable drug. Among the supporting interventions that AMFm requires are “interventions to expand ACT access to poor people, especially the poorest quintile, and other vulnerable groups” [11]. How can such populations best be reached?

In a 2008 study in Mali, MSF found that even when the public sector provided ACTs free of charge, other fees (e.g., for consultation or lab tests) could drive up costs to make ACT treatment inaccessible [22],[24]. In Bo District, Sierra Leone, the cost of health care per disease episode is 25 working days [24].

Furthermore, affordable health care does little good when it is not geographically accessible. This issue is particularly relevant for malaria, which can kill a child within 24 hours after the onset of symptoms. In a 2008 study of malaria treatment projects in Chad, Mali, and Sierra Leone, MSF found that access to malaria treatment improved dramatically only after the implementation of decentralized delivery models relying on trained MVWs to reach rural areas, *combined with* decreasing or abolishing user fees for health services [24]. As an extension of the public health system, the MVWs learned how to use RDTs, provide ACTs for confirmed malaria cases, and refer negative and severe cases to health centers. Findings from other contexts, including Cambodia [3] and Eritrea [25], suggest that community health workers (CHWs) can be an effective means of reaching remote or marginalized populations, particularly for relatively straightforward interventions.

However, many questions remain regarding optimal management of CHWs or MVWs, including how best to pay, retain, train, supervise, and incorporate them into the formal health system, how to maximize service uptake by the population, and how to scale up to national level [26]–[29]. Nevertheless, the question of how to reach patients in remote areas remains urgent: the lack of geographical access to public health centers was a primary rationale for subsidizing ACTs in private outlets, in hopes that they would better reach remote areas; however, pilot projects indicate that uptake of subsidized ACTs in remote rural private sector outlets remained significantly lower than in population centers [30]. Comparing the relative efficacy of MVWs versus private sector subsidies in bringing affordable ACTs to remote areas should be a priority research question.

Indeed, operational research and monitoring and evaluation efforts (M&E) will be critical in AMFm's Phase 1. AMFm is encouraging countries to submit funding requests for M&E and operational research, and plans to carry out a multi-country evaluation as well [11]. However, up to this point, much of the policy debate and research has focused either on the AMFm's potential impact on the emergence of resistance, and/or its impact on price. Of equal if not greater concern are health outcomes. The application guidelines encourage countries to monitor ACT availability, price, market share, and barriers to access, but do not mention health outcomes. Understanding the extent to which a subsidy meets patient needs will require data on incidence of uncomplicated malaria, severe malaria, and mortality attributed to malaria, as well as on ACT coverage, levels of treatment literacy and adherence, and ability to pay. As countries implement various supporting interventions, it will be critical to include patient-centered indicators in monitoring efforts.

## II. Securing Artemisinin Supply and Removal of Artemisinin Monotherapy

Several other measures could improve the AMFm's efficacy. First, the AMFm should take steps to avoid repeating the global artemisinin shortages in 2004–2005 that wreaked havoc on treatment programs [31]. Figure 1 indicates both how widely artemisinin raw materials prices have fluctuated, and how lower raw materials prices combined with generic competition have reduced ACT prices. Current estimates indicate a 40-ton shortfall of the artemisinin starting material needed to produce 240 million treatments in 2010 [6]. The 2010 availability depends on what is being planted by farmers today, due to the 14-month time span required from seed to finished product. Furthermore, the highly volatile food crop market impacts artemisinin supply, and farmers' decisions on whether to plant *Artemisia annua* are subject to imperfect information—thus, we cannot expect that market forces alone will guarantee sufficient supply in the short term. The malaria community has moved too slowly to stabilize the market. For example, in January 2009 the UNITAID Board delayed approval of a revolving fund for this purpose, meaning that the planting period for Asian farmers was missed. The AMFm should act as quickly as possible to ensure a sufficient, stable artemisinin supply.

Second, countries should use regulatory measures to implement the WHO ban on artemisinin monotherapy and to remove chloroquine (CQ) as a treatment option for *P. falciparum*. Country experience shows that subsidized ACTs only have a limited effect in crowding less effective antimalarials out of the market [3],[30]. Not long after WHO first called on manufacturers to stop marketing artemisinin monotherapy, 40 out of 74 identified manufacturers announced their cooperation; today, 35 of 77 endemic countries either do not allow the marketing of artemisinin monotherapy or plan to disallow it soon [2],[32]. A combination of economic, regulatory, and enforcement tools should be used to remove artemisinin monotherapy and ineffective antimalarials from the market.

## III. Implications for Public and Private Sector Support

A central issue raised here and in debates preceding the AMFm is how to calibrate international support for the public and private sectors. Patient use of each sector differs substantially by country. For example, in Cambodia 80%–90% of people seek care for fever outside the public health system, while in Senegal 75% do so through the public sector [3],[30],[31].

Evidence from pilot projects implementing private sector ACT subsidies reflects wide variance between countries in each sector's capacity. For example, a pilot subsidy in Tanzania translated into significantly lowered prices and increased use of ACT through the private sector. In contrast, in Cambodia, the publicly supported MVW program was far more successful in increasing access to appropriate treatment compared to the private sector–based social marketing of subsidized RDTs and ACT co-blisters [30].

While pilots indicate that the AMFm *can* improve access through the private sector, the initiative should complement rather than undermine public sector efforts, particularly since some policies are far easier to implement in the public sector, including: using RDTs, providing free care to the vulnerable, regulating trained health staff, and avoiding perverse incentives to overprescribe. It will be important to monitor for any potential negative effects the AMFm could have on public ACT provision, particularly in countries with high levels of public sector usage. Weakening of public sector capacity could occur for many reasons, including: decreased international funding, competition with the private sector for limited ACT stock (of particular concern if raw material shortages recur), declining political attention to improving public service provision, and/or competing demands on limited managerial and administrative resources in national malaria control programs. Currently, the guidelines put low priority on expanding ACT availability in the public sector; the AMFm should clarify that countries may use the increased affordability of ACTs to supply both the private and public sectors.

## IV. Conclusions

The AMFm is an innovative but untested global initiative with the potential for both positive and unintended consequences for health. Keeping the focus on quality care—through patient-centered policies on drug choice, diagnostics, delivery, and M&E—will help the AMFm to meet the long unfulfilled promise of artemisinin for the millions who continue to suffer from malaria today.

## Abbreviations

ACT: artemisinin-based combination therapy
AMFm: Affordable Medicines Facility–malaria
CHW: community health worker
CQ: chloroquine
FDC: fixed-dose combination
GFATM: Global Fund to Fight AIDS, Tuberculosis and Malaria
M&E: monitoring and evaluation
MSF: Médecins Sans Frontières
MVW: malaria village worker
RDT: rapid diagnostic test
SP: sulfadoxine-pyrimethamine
WHO: World Health Organization
WHO-PQ: WHO-prequalified
